# Atypical Presentation of Dysphagia in a Patient Diagnosed Later With Dermatomyositis: A Case Report

**DOI:** 10.7759/cureus.19964

**Published:** 2021-11-28

**Authors:** Ali Elmdaah, Abuobeida Ali, Zulakha Nadeem, Mohamed Habieb, John Pradeep, Kevin Metangi

**Affiliations:** 1 Gastroenterology, Peterborough City Hospital, Peterborough, GBR; 2 Rheumatology, Peterborough City Hospital, Peterborough, GBR

**Keywords:** gottron's sign, dermatomyositis, diabetes mellitus, myositis, dysphagia

## Abstract

Dysphagia has been reported in 10%-73% of patients with dermatomyositis. We present the case of a 58-year-old female patient who presented to the emergency department of Peterborough City Hospital with acute-onset difficulty in swallowing. Physical examination demonstrates proximal muscle weakness of the upper limbs and symmetrical skin rash over the face, chest, and thighs. Both clinical and laboratory findings pointed towards the diagnosis dermatomyositis. Oesophagogastroduodenoscopy identified no significant abnormality reducing the possibility of dysphagia due to an intrusive lesion, such as an abscess or a malignancy. MRI scan of the lower limbs revealed evidence of proximal myositis. CT neck, chest, abdomen and pelvis exclude any associated malignancy. The patient was treated initially with intravenous pulses of methylprednisolone for three days, and then switched to oral prednisolone and cyclophosphamide cycles and was considered for intravenous immunoglobulins as her symptoms had not completely resolved.

## Introduction

Dysphagia has been frequently reported in patients with inflammatory myopathy and 10%-73% of patients with dermatomyositis developed weakness of oropharyngeal, laryngeal, and oesophageal musculature that leads to impairment in the oropharyngeal phase of swallowing [1-3]. The proper mechanism of dysphagia in patients with dermatomyositis remains unclear and it is thought to be due to impaired muscle contractions and decreased hypo-laryngeal excursion that leads to impaired relaxation of the upper oesophageal sphincter [4]. There are literatures that have demonstrated increased frequency of dysphagia in dermatomyositis patients with internal malignancy in comparison with patients without malignancy [5]. This risk of developing cancer can be extended to up to five years [6]. Delayed treatment, bulbar involvement, respiratory involvement, old age, and occurrence of malignancy are associated with poor prognosis [7].

## Case presentation

A 58-year-old Caucasian woman presented with an acute worsening of dysphagia over 48-72 hours. She had noticed gradual worsening of symptoms over a three-week period with choking on attempt to ingest and had stopped taking in neither solid nor liquids in her diet. She came in with no significant weight loss, apart from that noted in the three days prior to emergency department attendance.

**Figure 1:**
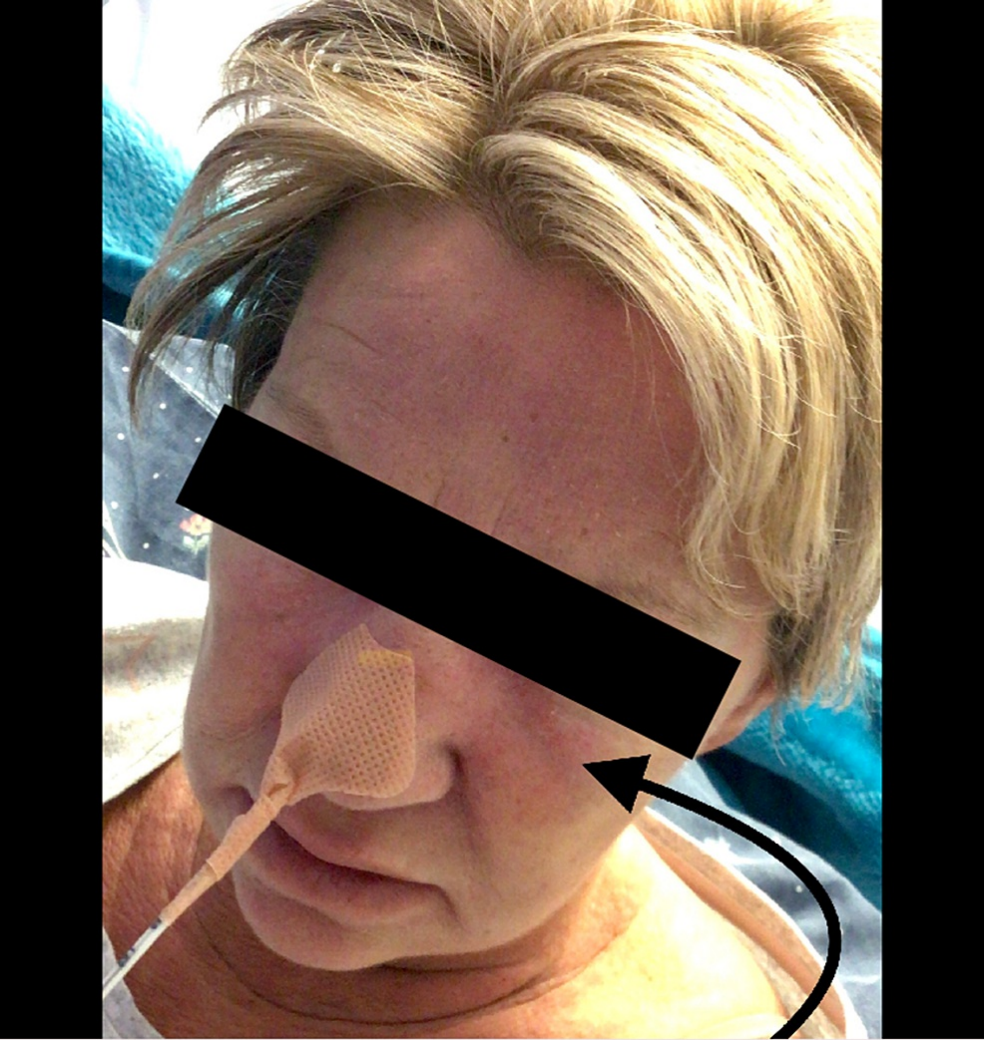
Violet pink coloured oedematous rash over the face

**Figure 2:**
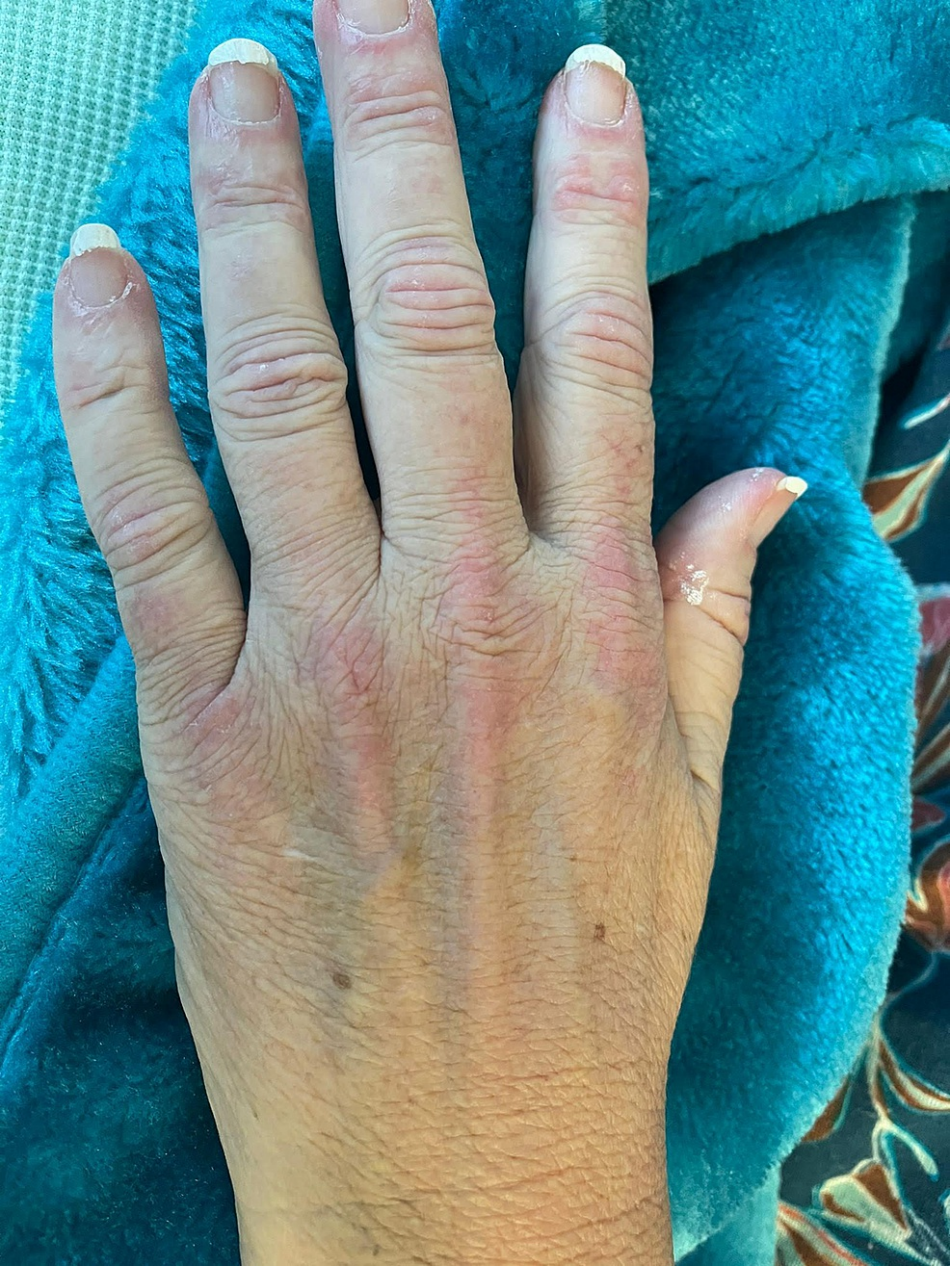
Gottron's sign: violet coloured bumps over the knuckles (metacarpophalangeal joints) of the patient's left hand

In her history she further indicated that she had travelled from Spain three months prior and had developed a rash after eating shellfish which was managed as an allergic reaction but with no relief of symptoms on antihistamines. The rash, however, progressed to affect face, chest, abdomen, and limbs (Figures 1, 2). Whilst in Spain she mostly lived in her home, and she neither went for camping nor spent her stay outdoors. She was otherwise normally fit and well with a background history of hypothyroidism and insulin-dependent diabetes mellitus with no known allergies.

On presentation, she had a rash affecting her face (forehead, cheeks, nose, and chin) and dorsum of hands - metacarpophalangeal joints and knuckles. She had a rough, dry violaceous rash affecting elbows and side of left thigh. She had no obvious cranial nerve deficit with motor power 5/5 in four limbs with no obvious neurological deficits and had normal coordination. Oral examination was unremarkable. Flexible naso-endoscopy showed that nasopharynx, oropharynx, and vocal cords were normal, and neck examination showed no obvious palpable lump indicating cause of dysphagia was unlikely obstructive. Initial blood tests revealed C-reactive protein to be 8 (<5 mg/L), erythrocyte sedimentation rate 20 (0-18 mm/h), and creatine kinase (CK) 335 U/L (normal range: 25-200 U/L). Urgent computed tomography scan of the neck, chest, abdomen, and pelvis and esophagogastroduodenoscopy identified no significant abnormality, reducing the possibility of dysphagia due to an intrusive lesion, such as an abscess or a malignancy [8].

Further review by Dermatology and Rheumatology team identified progressive muscle weakness predominantly in proximal muscles with no breathing difficulty with proximal muscle weakness of MRC grade 4 predominantly in upper limbs bilaterally with normal neck extension power. She had a heliotropic rash over face and raised erythema spreading to the chest and limbs. Gottron's papules were noted on the hands and periungual erythema with abnormal nailfold capillaries (Figure 2).

**Figure 3:**
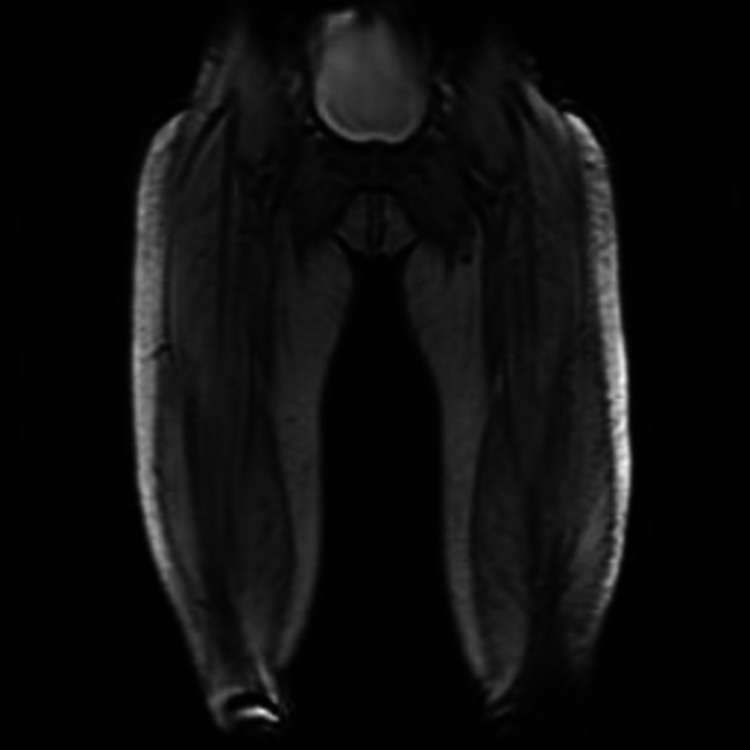
MRI scan lower limb showed proximal muscle myositis

Swallow assessment identified reasonable oral motor skills bilaterally with limited jaw opening; however, oral mucosa was clean and moist, indicating that she was managing her secretions, vocal quality was good, and she was able to trigger dry swallow reflex with effort, some laryngeal elevation was palpable but weak, and all these indicated a pharyngeal phase dysphagia. Repeat serum CK levels further increased to 391 U/L (normal range: 25-200 U/L) after 48 h and lactate dehydrogenase (LDH) was also raised. Antinuclear antibodies (ANAs) were positive (>200), as well as ANA by Hep 2 with speckled staining pattern and anti-Jo antibodies were negative with positive anti Ro with negative antineutrophil cytoplasmic antibodies, HMGCoA reducatse antibodies, and dsDNA. Myositis antibodies SAE-1 and Ro-52 were positive. Urgent MRI of proximal muscles showed active, proximal myositis of both lower limbs (Figure 3). These clinical findings and the results of laboratory tests supported a diagnosis of dermatomyositis.

Treatment with intravenous (IV) methylprednisolone pulses was urgently commenced with introduction of high-dose prednisolone at 1 mg/kg (60 mg once daily orally for four weeks) [9]. She was then commenced on cyclophosphamide cycles as EUROLUPUS regimen 500 mg IV infusion for total six infusions every two weeks initially to minimise the long-term risk of malignancy as it increases in dermatomyositis. She was also given MESNA 2 h before and 6 h after the cyclophosphamide infusion at a dose of 400 mg orally twice daily on days of cyclophosphamide infusion to decrease the risk of haemorrhagic cystitis. Her bloods including full blood count, urea and electrolytes, and LDH were monitored at day 2 and day 10 post-infusion. She then showed some improvement of dysphagia, muscle weakness, and laboratory parameters [10,11]. She was commenced on nasogastric feeds with continued rehabilitative physiotherapy and dysphagia therapy including oropharyngeal exercise with further assessment of swallow via video-fluoroscopy or fiberoptic endoscopic evaluation of swallowing and muscle biopsy.

## Discussion

Dermatomyositis is one of the inflammatory myopathies caused by complement (C5b-9)-mediated microangiopathy that affects the skin and muscles. It has a characteristic skin manifestation of heliotrope rash over the upper eye lid, flat red rash over the face, and Gottron's papules on the knuckles (Figures 1, 2). 

Although dysphagia is the frequently reported associated symptom with dermatomyositis, the severity of the dysphagia as first presentation to patient with later diagnosed with dermatomyositis has been rarely reported. The challenging point in our case study is history taking as the main concern of the patient's primary presentation was dysphagia without reporting muscle weakness [12]. In our case the patient could not even swallow her saliva without evidence of disease progression like progressive muscle weakness or worsening inflammatory markers or CK level. In this case, Gastroenterology, Rheumatology, Nutrition, and Speech and Language therapy were all involved in her hospital admission care. As there was a reported association between malignancy and dermatomyositis, we did CT scan of the neck, chest, abdomen, and pelvis and that revealed no cancer. Oesophagogastroduodenoscopy identified no significant abnormality. We have reached the diagnosis of dysphagia as acute presentation of dermatomyositis after all other possible causes of dysphagia had been ruled out.

Glucocorticoids remain the main initial available treatment. The addition of immunosuppression is required for disease control. Prior reports described patients with dermatomyositis who did not respond to steroids and who had required IV immunoglobulins [13]. In our case the patient had responded to steroids and showed initial improvement in swallowing a week after commencing immunosuppressive therapy with improvement in muscle strength and laboratory parameters. However, her dysphagia did not completely improve and she was considered for IV immunoglobulins infusion. 

A multidisciplinary approach is necessary to diagnose severe acute dysphagia due to underlying dermatomyositis rather than other structural gastroenterological or neurological causes. Also, it is important to perform neurological examination to examine the proximal muscles, then to think about the associated diseases with the primary patient's concern. Appropriate supportive care is important because dysphagia can be life-threatening and last for a long time. 

## Conclusions

We have reported an acute presentation of dysphagia as the main symptom of patient presentation due to underlying dermatomyositis. Although dysphagia is a known complication of dermatomyositis, sudden onset of dysphagia without the notable aggravation of other symptoms can make the diagnosis and treatment challenging. We have showed the importance of taking accurate history and performing relevant systematic examination for all patients who presented with acute difficulty in swallowing. This also highlights the importance of neurological examination of proximal muscles, then to think about the associated diseases with the primary patient's concern.

## References

[1] Oh TH, Brumfield KA, Hoskin TL, Stolp KA, Murray JA, Bassford JR (2007). Dysphagia in inflammatory myopathy: clinical characteristics, treatment strategies, and outcome in 62 patients. Mayo Clin Proc.

[2] Williams RB, Grehan MJ, Hersch M, Andre J, Cook IJ (2003). Biomechanics, diagnosis, and treatment outcome in inflammatory myopathy presenting as oropharyngeal dysphagia. Gut.

[3] Ebert EC (2010). Review article: the gastrointestinal complications of myositis. Aliment Pharmacol Ther.

[4] Langdon CP, Mulcahy K, Shepherd KL, Low VH, Mastaglia FL (2012). Pharyngeal dysphagia in inflammatory muscle diseases resulting from impaired suprahyoid musculature. Dysphagia.

[5] Ponyi A, Constantin T, Garami M (2005). Cancer-associated myositis: clinical features and prognostic signs. Ann N Y Acad Sci.

[6] Behan W, Sturrock R (2004). Idiopathic inflammatory myopathies. Reports on the rheumatic diseases series 5; topical reviews.

[7] Cordeiro A, Isenberg D (2006). Treatment of inflammatory myopathies. Postgrad Med J.

[8] Azuma K, Yamada H, Ohkubo M, Yamasaki Y, Yamasaki M, Mizushima M, Ozaki S (2011). Incidence and predictive factors for malignancies in 136 Japanese patients with dermatomyositis, polymyositis and clinically amyopathic dermatomyositis. Mod Rheumatol.

[9] Dagan A, Markovits D, Braun-Moscovici Y, Rozin A, Toledano K, Balbir-Gurman A (2013). Life-threatening oropharyngeal aphagia as the major manifestation of dermatomyositis. Isr Med Assoc J.

[10] Choy E, Hoogendijk J, Lecky B, Winer JB (2005). Immunosuppresants and immunomodulatory treatment for dermatomyositis and polymyositis. Cochrane Database Syst Rev.

[11] Ramachandran RB, Swash M (2004). Pharyngeal dysphagia in dermatomyositis: responsive to cyclophosphamide. J Clin Neuromuscul Dis.

[12] Iannone F, Giannini M, Lapadula G (2015). Recovery of barium swallow radiographic abnormalities in a patient with dermatomyositis and severe dysphagia after high-dose intravenous immunoglobulins. J Clin Rheumatol.

[13] Marie I, Menard JF, Hatron PY (2010). Intravenous immunoglobulins for steroid-refractory esophageal involvement related to 39 polymyositis and dermatomyositis: a series of 73 patients. Arthritis Care Res (Hoboken).

